# Detailed analysis of total colectomy on health-related quality of life in adult patients with ulcerative colitis

**Published:** 2017

**Authors:** Hamid Asadzadeh Aghdaei, Faranak Ghasemi, Mina Nooraliee, Mohammad Sadegh Fazeli, Anaraki, Dario Sorrentino, Hedieh Balaii, Shabnam Shahrokh

**Affiliations:** 1 *Basic and Molecular Epidemiology of Gastrointestinal Disorders Research Center, Research Institute for Gastroenterology and Liver Diseases, Shahid Beheshti University of Medical Sciences, Tehran, Iran*; 2 *Department of surgery, Imam Khomeini Medical complex, Tehran University of Medical Sciences, Tehran, Iran*; 3 *Colorectal Division of Department of Surgery, Taleghani Hospital, Shahid Beheshti University of Medical Sciences, Tehran 19835-178, Iran*; 4 *IBD Center, Department of Medicine, Division of Gastroenterology, Virginia Tech Carilion School of Medicine, Roanoke, VA, USA*; 5 *Gastroenterology and Liver Diseases Research Center, Research Institute for Gastroenterology and Liver Diseases, Shahid Beheshti University of Medical Sciences, Tehran, Iran*

**Keywords:** Colectomy, Quality of life, Ulcerative colitis

## Abstract

**Aim:**

The aim of this study was to explore the quality of life (QoL) in a group of patients who had an intractable disease on medical therapy including biologics and underwent surgery.

**Background:**

Quality of life of patients with ulcerative colitis (UC) has been measured with a series of multiple questionnaires

**Methods:**

An observational cross sectional study was carried out on 68 patients with documented UC referring to an IBD clinic in a tertiary hospital. Patients with UC who had a colectomy because of intractable disease and were in remission for a year were eligible for enrollment Patients were instructed to fill the SF-36 Questionnaire (interviewer-administered) regarding quality of life. Side effects were evaluated with another questionnaire. Results were compared with the normal population of the community.

**Results:**

In comparison with normal population, patients having colectomy have better general QoL. Impotency and incontinency were most common adverse events after colectomy while the adverse events that decreased the QoL significantly were anal secretions and number of bowel movements per day without using antidiarrheal- drugs.

**Conclusion:**

In conclusion, our study showed a significant improvement of general QoL in a selected group of UC patients, who were in clinical remission following IPAA and only number of bowel movements per day and anal secretions significantly impaired their QoL. We suggest that a disease-speciﬁc questionnaire should be designed, making changes in health-related QOL more detectable over time, since it is more sensitive to these changes in IBD patients than a general questionnaire.

## Introduction

 Treatment of ulcerative colitis (UC) relies on modifying immune system and done by a step-up approach according to the severity and the spread of disease, which starting with 5-ASA drugs in milder and more limited disease to adding corticosteroids, immunomodulators and biologic drugs in more severe and extensive type. Colectomy would be carried out in intractable cases and emergencies (1-2). Although medical therapy is the basis of UC treatment, surgical management still have an important role since it can be an option for cases that are resistant to medical treatment, premalignant or malignant lesions and even be lifesaving in emergent cases (3,4). In comparison to the past, the role of surgeries has changed from decreasing mortality to become an option for reducing morbidity and especially improving quality of life (QoL) (5). Most of the UC patients, experience some degrees of impairment in health-related quality of life (HRQL) compared to the general population (6). One of the essential aspects of treatment in these patients is evaluation of QoL and disability assessments associated with medical or surgical therapies. The World Health Organization’s definition for quality of QoL is “the perception of the individual of his/her position in life in the cultural context and the value system in which he/she lives in relation to his/her objectives, expectations, standards, and concerns” (7). Recently the QoL of these patients has been measured with a series of multiple questionnaires based on psychological and physical function, with a standard and quantitative procedure (6). The aim of this study was to explore QoL in a group of patients who had an intractable disease on medical therapy including biologics and underwent surgery. 

## Methods

An observational cross sectional study was carried out on 68 adult patients (95% reliability and d=80%) with documented UC were consecutively recruited from a tertiary center. Patients were identified by retrospective chart review. All patients with UC who had a colectomy because of intractable disease and were in remission for a year, were eligible for enrollment. All of them had pouch reconstruction and patients with a (sub)total colectomy or ostomy were excluded. We chose patients in remission in order to evaluate their QoL, since those who are not in remission will surely have a low QoL. 

Patients were instructed to fill the Short Form-36 Questionnaire (interviewer-administered) regarding QoL. This questionnaire was translated in Persian and validated. Medical feedbacks based on SF-36 Questionnaire which contains 36 questions and 8 different invoice reviews was a general approach that has been used for measurement of the life quality in different populations and patients. It includes pain, general health, mental health, physical function, mental function, and the role of emotions and physical power. Each scale has been rated by number of items: Physical functioning, Role limitations due to physical health, Role limitations due to emotional problems, Energy/fatigue, Emotional well-being, Social functioning, Pain and General health are recorded by 10, 4, 3, 4, 5, 2, 2 and 5 items respectively. On the other hand, two prime fields have been followed by SF-36 questionnaire, including mental (according to the emotional and mental items scoring 0-100) and physical (regarding to the functional system rating 0-50) QoL. This questionnaire has been graded from 0 to 100. A higher score shows better QoL. We also evaluate the types and frequency of adverse events after surgery. All surgeries were performed by two surgeons with one technique. This study was approved by Shahid Beheshti University of Medical Sciences ethics committee and written informed consent was obtained from each participant.

The Student’s t-test was used for comparisons of variable. SPSS statistical software version 19 (SPSS INC, Chicago, IL, United States) was used to perform statistical analysis. P < 0.05 was considered statistically significant. Continuous variables are expressed as means and standard deviation, and 95% CI as appropriate. 

## Results

**Data collection and Subject characteristics**

79 patients with UC were eligible for this study, of which were willing sixty eight to participate and therefore, enrolled in our study. All patients were outpatients. Each questionnaire was filled out completely by 100% of patients. Baseline characteristics are shown in Table 1. 

**Table 1:** Demographic characteristics of patients included. Results are expressed in means±SD and (%).

	Baseline characteristics
Age (Mean ± SD)	39.26±11.08
Age at Diagnosis (mean±SD)	29.71 ± 10.208
Age at colectomy (Mean ± SD)	37.55 ± 11.176
Gender	
male	37(56.9%)
female	28(43.1%)
BMI (kg/m^2^)	
under weight	8(12.3%)
normal	35(53.8%)
over weight	16(24.6%)
Obese	6(9.2%)
Married	47(72 %)

**Table 2:** The Quality of life scoring in colectomy patients, consisting of general, physical and mental QoL evaluation. The results are presented in means±SD and SF-36 values

QoL	Variables
General QoL Mean ±SD Range	86.73±13.89 44-100
Physical QoL Mean ±SD Range	30.83±6.50 10-39
Mental QoL Mean±SD Range	36.74±10.48 18-90

**Table 3:** Pearson correlation coefficient of bowel movements per day and quality of life in Ulcerative Colitis patients who have undergone colectomy

	Pearson correlation coefficient	P-value
General QOL	-0.240	0.044
Physical QOL	-0.222	0.060
Mental QOL	-0.342	0.004

**Table 4:** Relation between nocturnal defecation, stool incontinency, and anal secretions with quality of life in ulcerative colitis patients whom undergone colectomy

	Nocturnal defecation			Stool incontinency			Anal secretions		
	Number	Mean±SD	P-value	Number	Mean±SD	P-value	Number	Mean±SD	P-value
General QOL	21/70	86.48±11.84	0.733	26/70	88.27±9.31	0.171	12/70	81.42±10.79	0.0169
Mental QOL	21/70	37.14±8.53	0.836	26/70	37.19±7.88	0.785	12/70	39.08±16.71	0.400
Physical QOL	21/70	31.38±6.65	0.824	26/70	32.61±5.97	0.138	12/70	27.00±5.97	0.015

**Table 5:** Relation between enuresis and urinary hesitancy with quality of life in ulcerative colitis patients whom undergone colectomy

	Enuresis			Urinary hesitancy		
	Number	Mean±SD	P-value	Number	Mean±SD	P-value
General QOL	9/70	82.33±10.18	0.363	13/70	84.46±9.12	0.674
Mental QOL	9/70	34.55±5.80	0.507	13/70	39.15±16.90	0.362
Physical QOL	9/70	28.66±6.54	0.228	13/70	31.54±4.70	0.796

**Figure 1.**
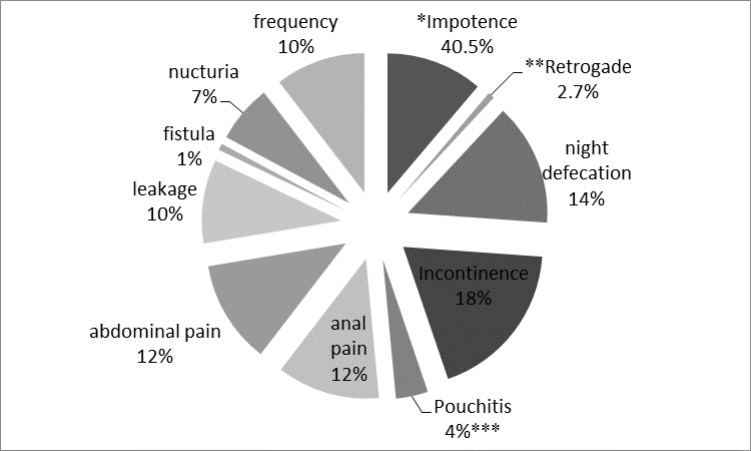
The percentage of adverse events after colectomy including impotence and retrograde which are evaluated in male; night defecation; incontinence; pouchitis; anal pain; abdominal pain; leakage; fistula; nucturia and the frequent defecation.

*, ** The percentage of impotence and retrograde have been calculated in patients underwent colectomy; *** Diagnosis of pouchitis was based on occurrence of certain symptoms in combination with endoscopic findings.

**Effects of colectomy on patients’ QoL with previous intractable disease**

According to SF-36 questionnaire values, general, mental, and physical aspects of QoL, the scores have been calculated in all patients. In Comparison to normal population colectomy patients have better general Qol but lower physical and mentally related QoL.

**Analysis of each entity of SF 36- questionnaire**

The questionnaire including six separate entities; General health: Rating the patients’ health in general compared to one year ago, limitation of activities during a typical day, physical health problems, emotional health problems, energy and emotions and social activities which data are shown in supplementary tables 1-**8**. 

**Frequency of adverse events after colectomy**

A significant association was observed between the number of defecation according to the following categories: less than one time=2.9%; 1-3 times=11.7%; 3-6 times =38.2% and more than 6 times per day=42% (p value: 0.043).

## Discussion

This study evaluated the QoL in patients who underwent colectomy because of intractable disease and were in remission phase during a past year. In the vast majority of other studies, the improvement of the QoL is defined as a statistically significant increase in the scoring of the questionnaires, but present evaluation gives an answer to part of daily life that is more affected by colectomy. Mostly, mean score of general QoL in UC patients after colectomy is higher than Iranian general population but with focusing on each section (mentally and physically) separately, mean score is lower in UC patients (8). The justification for this can be that patients had severe and intractable illness before surgery and large part of their problems disappear with surgery including the systemic inflammation, abdominal pain and bleeding. So general QoL is much better than a healthy individual who spend his/her monotonous life but actually in detail cannot ignore the basic physical and mental problems. There is no exact data to compare these scores with Iranian UC patients on Anti TNF. Compare to international statistics of colectomy: Perceived and actual QoL among patients with mild, moderate, and severe UC were compare by Waljee et al; patients who had undergone colectomy for UC; and a control group of healthy patients. UC patients without colectomy had a significantly higher median utility than post-colectomy patients. Those who had undergone colectomy viewed the scenario describing life with moderate UC significantly less favorable than UC patients on medical therapy (9). Fazio et al showed a good QoL in 93% of their 645 patients studied for 40 months after colectomy and they concluded that the presence of a temporary ileostomy did not seem to influence the QoL (10).

In addition, each part of questionnaire analyzed with more details to asses which aspects of QoL including General health: Rating the patients’ health in general compared to one year ago, limitation of activities during a typical day, physical health problems, emotional health problems, energy and emotions and social activities that in comparison with Iranian general population emotional role functioning ,social role functioning and mental health problems was significantly different between two groups in favor of colectomy patients.

Impotency and incontinency were most common adverse events after colectomy while the adverse- events that decreased the QoL significantly were anal secretions and number of bowel movements per day without using of antidiarrheal- drugs such as Loperamide.

A population-based cohort on Norwegian IBD patients found lower SF-36 scores for almost every dimension in the IBD group compared with healthy participants. The main differences were related to general, physical, and emotional health. They also found a relation between severity of IBD and health-related QoL, were general health, bodily pain, and role-physical dimensions were most affected (7). Casellas et al. revealed significantly lower scores in all dimensions in active IBD patients in contrast with those in remission. Disease activity, time of evolution since diagnosis, and female sex had a worse health-related QoL. The type of IBD or geographical area of residence did not influence QoL (6). A placebo-controlled double blinded study evaluated the impact of infliximab on health-related QoL in UC patients. They concluded that a sustained significant benefit till a year was seen in patients with UC on maintenance infliximab (11). Another population-based study in Swedish patients with IBD showed that patients with UC had a higher level of health-related and disease-specific QoL compared with patients with Crohn’s disease. An ileostomy did not seem to affect their patients’ QoL, but an ileoanal anastomosis did by reducing it (12). Heikens and Francone et al. reported increased and almost normal QoL in patients undergoing ileal pouch anal anastomosis (13,14).

The QoL in patients with severe UC after total colectomy versus cyclosporine was compared by Cohen et al. The latter group reported a better stool consistency, better sleep, less abdominal and rectal pain, and less trips to the toilet (15). Early IPAA in severe UC reduced health costs and had an equivalent QoL compared to extensive medical therapy (16). Van der Valk et al. conducted a large cohort from the COIN study and their results revealed that health care costs were mainly due to medications, whereas hospitalization and surgery were minorities (17).

In conclusion, our study showed a significant improvement of general QoL in a selected group of UC patients, who were in clinical remission following IPAA and only number of bowel movements per day and anal secretions were significantly impaired their QoL.

Our study had limitations including a retrospective design. Retrospective designs make way for prospective designs such as cohorts, as they are relatively inexpensive and time consuming. Regarding, the timing of the study after treatment protocols, since all patients in both groups were in complete remission, we believe this should not be an important confounding factor.

We suggest that a disease-speciﬁc questionnaire should be designed, making changes in health-related QoL more detectable over time, since it is more sensitive to these changes in IBD patients than a general questionnaire. It should be emphasized that using both a disease-specific and general QoL questionnaire could fill the pitfalls of each other.
